# Efficacy of different forms of Guizhi Fuling Wan on reproduction and metabolism in women with polycystic ovary syndrome: A protocol for a meta-analysis of randomized controlled trials

**DOI:** 10.1097/MD.0000000000022954

**Published:** 2020-10-30

**Authors:** Min Liu, Hongqiu Zhu, Xiaodan Hu, Ying Zhu, Haiyan Chen

**Affiliations:** a Chengdu University of Traditional Chinese Medicine; b Department of Gynaecology, School of Medical and Life Sciences, Chengdu University of Traditional Chinese Medicine/Reproductive & Women-Children Hospital of Chengdu University of Traditional Chinese, Chengdu City, Sichuan Province, China.

**Keywords:** Guizhi Fuling Wan, metabolism, polycystic ovary syndrome, protocol, reproduction

## Abstract

**Background:**

Polycystic ovary syndrome (PCOS), is a common endocrine disorder in women characterized by increased androgen levels, ovulatory dysfunction, and polycystic ovaries. Western medicine is widely used for the treatment of PCOS, but patient satisfaction is low, largely due to its associated gastrointestinal symptoms of nausea and diarrhea. Guizhi Fuling Wan (GFW) is a traditional Chinese medicine used to remove blood stasis and dissipate phlegm for treating gynecological diseases that was invented by Zhang Zhongjing in the Eastern Han dynasty. In recent years, GFW has been widely used to treat patients with PCOS. This study aims to assess the efficacy and safety of GFW in the treatment of PCOS through a systematic review and meta-analysis.

**Methods:**

All randomized controlled trials connected with GFW targeting PCOS will be searched in the following electronic bibliographic databases from their earliest recorded publications to December 2020 without any language restrictions: MEDLINE, Embase, PubMed, Web of Science, China National Knowledge Infrastructure, Chinese Biological Medicine Database, Wan-fang data, Chinese Technical Periodicals, and other databases. The primary outcomes include Sex hormone levels, ovulation rate, pregnancy rate, and total effective rate. The secondary outcomes were Total cholesterol, triglyceride, low-density lipoprotein, high-density lipoprotein, fasting glucose, fasting insulin, insulin sensitivity index, body mass index, hypertrichosis score, acne score, adverse reactions, etc. Two reviewers will independently conduct cations retrieval, de-duplication, filtering, quality assessment, and data analysis by Endnote X9.1 and Review Manager software (RevMan V.5.3). Meta-analysis and/or subgroup analysis will be performed on the included data.

**Discussion:**

This study will investigate the application of GFW in the treatment or prevention of PCOS, and provide a high-quality synthesis to judge whether GFW is an effective and safe intervention for PCOS.

**PROSPERO registration number:**

CRD42020192405

## 1 Introduction

Polycystic ovary syndrome (PCOS) is a common reproductive endocrine disease characterized by menstrual disorder, oligo/anovulation, hyperandrogenemia, polycystic ovaries, and insulin resistance.^[1]^ Studies^[2,3]^ show that PCOS with complex etiology affects 6% to 15% women of childbearing age, approximately 75% of whom experience infertility due to anovulation. The pathogenesis of PCOS is mostly metabolic disorder caused by insulin resistance and secondary hyperinsulinemia, which can lead to endometrial cancer, cardiovascular disease, type 2 diabetes, and other diseases over time, affecting women's physical, and mental health.^[4–6]^ Metformin is considered an insulin sensitizer as it enhances signaling through the insulin receptor, resulting in decreased IR, but patient satisfaction is low, largely due to its associated gastrointestinal symptoms of nausea and diarrhea.^[7,8]^ Therefore, more and more PCOS patients are seeking treatment from complementary and alternative medicine, including Chinese medicine.^[9]^

Chinese herbal formula Guizhi Fuling Wan (GFW), originated in the Eastern Han Dynasty. Today, they are gradually becoming a prevalent treatment for PCOS in China. The formula is made up of the following 5 herbs: Cinnamomum cassia (L.) J. Presl (Guizhi), Poria cocos (Schw.) Wolf (Fuling), Paeonia veitchii Lynch (Chishao), Prunus persica (L.) Batsch (Taoren), and Paeonia suffruticosa Andr. (Mudanpi). Previous Studies have found that GFW may play an important role in treating PCOS-IR by acting on protein targets and pathways related to hormone regulation, inflammation, and immunity.^[10–12]^ Meanwhile, GFW can not only effectively reduce fasting blood glucose and fasting insulin, correct hypoadiponectinemia to improve insulin resistance and abnormal lipid metabolism,^[13]^ but also shorten the menstrual cycle, restore ovulation, and improve the pregnancy rate.^[14,15]^

At present, there is no relevant systematic evaluation and meta-analysis. Therefore, this study will assess the efficacy and safety of GFW for PCOS by summarizing the current evidences and conducting a meta-analysis, to further provide instructions for medical researchers and clinical doctors.

## 2 Methods

### 2.1 Study registration

The protocol is reported in accordance with the guide book of Preferred Reporting Items for Systematic Reviews and Meta-Analyses (PRISMA) Protocols.^[16]^ The protocol has been registered in the PROSPERO (CRD 42020192405) on 16 June 2020.

### 2.2 Search strategy

MEDLINE, Embase, PubMed, Web of Science, China National Knowledge Infrastructure, Chinese Biological Medicine Database, Wan-fang data, Chinese Technical Periodicals, and other databases will be searched from their earliest recorded publications to December 2020. The search results will be limited to human studies only, and all included studies were randomized controlled trial (RCT) without any language restrictions. Besides, the reference lists of the selected studies and published systematic reviews will be screened for additional studies. According to intervention, patients, and study type, the following key search terms are used: ([“Polycystic Ovary Syndrome” OR “Polycystic Ovarian Syndrome” OR “PCOS”] AND [“Guizhi Fuling” OR “gui zhi fu ling” OR “guizhifuling”] AND [“randomized” OR “randomly” OR “randomized controlled trials” OR “clinical trial”]). The search strategy in PubMed is shown in Table 1. Combinations of Medical Subject Headings and text words will be used. The search strategy will follow the PRISMA guidelines.

**Table 1:**
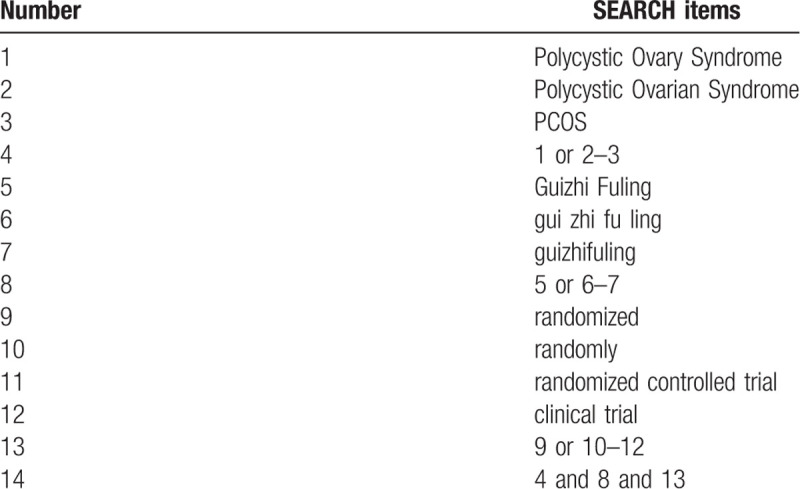
Search strategy for the PubMed database.

### 2.3 Inclusion criteria

#### 2.3.1 Type of study

Studies correlated to RCT and credible clinical observation without randomization due to emergency will be included. Due to language limitations, we searched Chinese and English articles to obtain more true and objective evaluation. All included articles were RCT. If an experiment does not explain randomization, the article will be considered as high risk in random sequence generation.

#### 2.3.2 Types of participant

Patients diagnosed with Polycystic Ovary Syndrome.

#### 2.3.3 Type of intervention

(1) GFW or capsule or decoction;
(2) GFW or capsule or decoction +Western medicine (the western drug regimen must be consistent with the control).

#### 2.3.4 Type of comparators

Western medicine.

#### 2.3.5 Types of outcomes

Primary outcomes were Sex hormone levels, such as testosterone, luteinizing hormone, follicle stimulating hormone, LH/FSH, 17β-estradiol, prolactin, ovulation rate, pregnancy rate, total effective rate. Secondary outcomes were Total cholesterol, triglyceride, low-density lipoprotein, high-density lipoprotein, fasting glucose, fasting insulin, insulin sensitivity index, body mass index, hypertrichosis (F-G) score, acne score, adverse reactions, etc.

### 2.4 Exclusion criteria

(1) Incorrect or duplicate data or data that cannot be extracted after contacting the corresponding author;
(2) The full text is not available upon contact with the corresponding author.

### 2.5 Data collection

#### 2.5.1 Data management

Endnote X9.1 will be used to manage and filter search citations, and the extracted data will be analyzed and synthesized by the Review Manager software (RevManV.5.3) (Cochrane Collaboration).

#### 2.5.2 Data extraction

**Figure 1:**
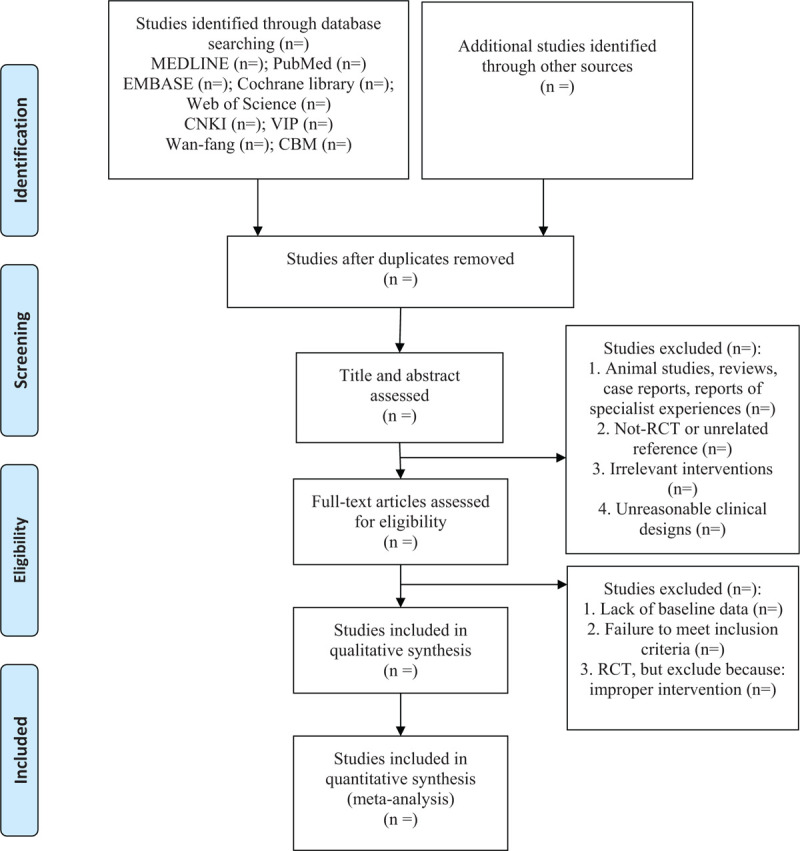
Preferred Reporting Items for Systematic Reviews and Meta-Analyses flow diagram of the study process.

PRISMA flowchart was used to show the citation selection process of the study (Fig. 1). Before retrieving the citation, all authors will discuss and determine the screening criteria. After the screening requirements were clear, the citations will be screened independently by the 2 authors, they will extract the data using standardized data extraction form and any differences of opinion between them will be resolved through discussion, if failed, by arbitration by the third author. We identified the following information for each trial:

1. The basic information of the article: the title of article, first author, year of publication, language.
2. Inclusion and exclusion criteria.
3. The baseline of the study: the sample size, age.
4. Interventions in the observation group and the control group such as drug name, dosage form, dose, frequency, course of treatment, etc.
5. outcome measures (If the unit of measurement included in the observation index is different, it will be converted into a unified unit).

For publications with insufficient data, we will try to obtain the missing data from the author.

#### 2.5.3 Risk of bias assessment

In this study, 2 authors independently evaluate the risk of bias using Cochrane's Risk-of-bias Tool, and generate a bias risk graph. It includes the following 6 aspects:

(1) random sequence generation,
(2) allocation concealment,
(3) blinding of participants, personnel and outcome assessment,
(4) incomplete outcome data,
(5) selective outcome reporting,
(6) other bias.

It visually analyzes the quality of literature, using greens, yellows and reds, as well as “+”, “-”, “?” The symbol stands for “low risk bias”, “high risk bias” and “unclear” to evaluate the risk of each included article from the above 6 aspects. Funnel plots will be created to assess the reporting bias. Dissymmetry funnel plot indicates high risk of reporting bias, while symmetric funnel plot indicates low risk. After the evaluation, cross-check will be carried out. If there are differences between the 2 authors, the results will be unified after mutual discussion or judged by the third author.

#### 2.5.4 Dealing with missing data

We try to ensure the integrity of data. As data in the included study may be lost, we will contact the corresponding author via email or telephone. If the missing data is still not available, the study will be excluded from the analysis.

### 2.6 Statistical analysis

#### 2.6.1 Data synthesis

RevMan V.5.3 is used for data analysis and quantitative data synthesis. For continuous data, if there is no heterogeneity, we will use mean difference (MD) or standard MD to measure the treatment effect of 95% CI. If significant heterogeneity is found, a random-effects model will be used. For dichotomous data, we will use a 95% Cl risk ratio for analysis (heterogeneity will be explained by text, as described below). In these trials, participants will be randomized into 2 intervention groups and individual measurements for each participant will be collected and analyzed. The results are represented as risk ratio for dichotomous data and standard MD for continuous data. The heterogeneity between studies was evaluated by the Q test and the I^2^ test. If the I^2^ test is less than 50%, a fixed effect model will be used for data synthesis; if the I^2^ test is between 50% and 70%, a random effect model will be used. If the I^2^ test is higher than 75%, we will look for possible causes from clinical and methodological perspectives, and provide a descriptive analysis or subgroup analysis.

#### 2.6.2 Subgroup analysis

If the heterogeneity is caused by clinical trials above-mentioned, for instance, treating with different dosage forms of GFW (GFW, Guizhi Fuling capsule and Guizhi Fuling decoction), subgroup analysis will be performed. And detailed subgroup will be classified according to the outcomes of data synthesis.

#### 2.6.3 Sensitivity analysis

When studies included after subgroup analysis have significant heterogeneity, a sensitivity analysis will be performed according to different study design, sample size, heterogeneity quality, methodological quality, and statistical approach to exclude studies with inferior quality and ensure the stability of analysis results.

#### 2.6.4 Assessment of reporting bias

In this analysis, if a sufficient number of studies (more than 10 trials) are included, publication bias and other reporting bias will be assessed by funnel plots.

#### 2.6.5 Quality of evidence

We will evaluate the quality of evidence for each outcome through the Grading of Recommendations Assessment, Development, and Evaluation system.^[17]^ Each outcome will be estimated in 5 dimensions: limitations, inconsistencies, indirectness, inaccuracy, and publication bias. And which will be classified into high, medium, low, or very low level.

#### 2.6.6 Ethics and dissemination

This study does not involve moral approval or ethical review, as we will use information from published studies. The final results will be published in a peer-reviewed journal based on PRISMA guidelines.

## 3 Discussion

Some articles have reported that GFW can effectively correct sex hormone disorder, improve PCOS insulin resistance, and play a positive role on ovulation and pregnancy.^[14,15]^ However, there is a lack of literature to evaluate the strength, safety and limitations of existing evidence as well as the effects of different dosage forms of GFW on PCOS endocrinology and reproduction. In order to optimize treatment for PCOS in women, we will systematically assess empirical evidence of GFW treating PCOS for the first time, and guided by the (PRISMA) statement and the recommendations of the Cochrane Handbook.^[18–20]^ The protocol may have some limitations, but if there are any significant revisions, we will update the PROSPERO records. The results of the systematic review will be published in peer-reviewed open access opinion journals to enable relevant scientific and clinical staff to obtain these results. The findings will also be disseminated in places where the target population is likely to be exposed. This analysis will explore the potential role of GFW in the treatment of COS-IR, hoping to provide convincing evidence for clinicians to make decisions.

## Author contributions

**Conceptualization:** Min Liu, Hongqiu Zhu.

**Data curation:** Haiyan Chen.

**Investigation:** Min Liu, Hongqiu Zhu, Ying Zhu.

**Methodology:** Min Liu, Hongqiu Zhu.

**Project administration:** Min Liu, Hongqiu Zhu, Xiaodan Hu.

**Writing – original draft:** Min Liu.

**Writing – review & editing:** Min Liu, Hongqiu Zhu.

## References

[1] Ding DC Tsai IJ Wang JH Coronary artery disease risk in young women with polycystic ovary syndrome. Oncotarget 2018;9:8756–64. 29492235 10.18632/oncotarget.23985 PMC5823557

[2] Fauser BC Tarlatzis BC Rebar RW Consensus on women”s health aspects of polycystic ovary syndrome (PCOS): the Amsterdam ESHRE/ASRM-sponsored 3rd PCOS consensus workshop group. Fertil Steril 2012;97:28–38. 22153789 10.1016/j.fertnstert.2011.09.024

[3] Li Y Ruan X Wang H Comparing the risk of adverse pregnancy outcomes of Chinese patients with polycystic ovary syndrome with and without antiandrogenic pretreatment. Fertil Steril 2018;109:720–7. 29525688 10.1016/j.fertnstert.2017.12.023

[4] Li Y Chen C Ma Y Multi-system reproductive metabolic disorder: significance for the pathogenesis and therapy of polycystic ovary syndrome (PCOS). Life Sci 2019;228:167–75. 31029778 10.1016/j.lfs.2019.04.046

[5] Rosenfield RL Ehrmann DA The pathogenesis of polycystic ovary syndrome (PCOS): the hypothesis of PCOS as functional ovarian hyperandrogenism revisited. Endocr Rev 2016;37:467–520. 27459230 10.1210/er.2015-1104 PMC5045492

[6] Rutkowska A Rachon D Bisphenol A (BPA) and its potential role in the pathogenesis of the polycystic ovary syndrome (PCOS). Gynecol Endocrinol 2014;30:260–5. 24397396 10.3109/09513590.2013.871517

[7] Facchinetti F Orru B Grandi G Short-term effects of metformin and myo-inositol in women with polycystic ovarian syndrome (PCOS): a meta-analysis of randomized clinical trials. Gynecol Endocrinol 2019;35:198–206. 30614282 10.1080/09513590.2018.1540578

[8] Kashani L Omidvar T Farazmand B Does pioglitazone improve depression through insulin-sensitization? Results of a randomized double-blind metformin-controlled trial in patients with polycystic ovarian syndrome and comorbid depression. Psychoneuroendocrinology 2013;38:767–76. 22999261 10.1016/j.psyneuen.2012.08.010

[9] Yuan Z Gu Q Discussion on classical prescriptions in the treatment of polycystic ovarian syndrome. Shanghai J Tradit Chin Med 2018;52:31–2.

[10] Shao X Clinical study of berberine combined with Guizhi Fuling Wan in patients with polycystic ovary syndrome with insulin resistance. Chin J Clin Res 2013;26:803–5.

[11] Cao L Zhang H Zhao Q Effects of Guizhi Fuling Wan combined with metformin on insulin resistation-related inflammatory response and oxidative stress response in PCOS patients. J Hainan Med Univ 2017;23:3211–4.

[12] Yu Y Zhang G Han T Based on network pharmacology and bioinformatics, analyze the mechanism of Guizhi Fuling Wan Bolus in the treatment of polycystic ovary syndrome. Chin J Pharmacol Toxicol 2019;33:832–3.

[13] Zhao Q Tan X Wang N Effect of Guizhi Fuling Wan on insulin resistance and adiponectin in rats with polycystic ovary syndrome. J N Chin Med 2012;44:116–7.

[14] Tao L Tuo A Liu M Effect of Guizhifu Ling Pill combined with berberine on patients with polycystic ovary syndrome with insulin resistance. Chin J Exp Tradit Med Formulae 2013;19:320–3.

[15] Wu J Deng H Liu Y Clinical research progress of Guizhi Fuling Wan in the treatment of infertility. J Guangzhou Univ Tradit Chin Med 2020;37:586–90.

[16] Shamseer L Moher D Clarke M Preferred reporting items for systematic review and meta-analysis protocols (PRISMA-P) 2015: elaboration and explanation. BMJ 2015;350:g7647. 25555855 10.1136/bmj.g7647

[17] Balshem H Helfand M Schunemann HJ GRADE guidelines: 3. rating the quality of evidence. J Clin Epidemiol 2011;64:401–6. 21208779 10.1016/j.jclinepi.2010.07.015

[18] Liberati A Altman DG Tetzlaff J The PRISMA statement for reporting systematic reviews and meta-analyses of studies that evaluate health care interventions: explanation and elaboration. J Clin Epidemiol 2009;62:e1–34. 19631507 10.1016/j.jclinepi.2009.06.006

[19] Moher D Liberati A Tetzlaff J Group P. Preferred reporting items for systematic reviews and meta-analyses: the PRISMA statement. Int J Surg 2010;8:336–41. 20171303 10.1016/j.ijsu.2010.02.007

[20] Higgins JPT Thomas J Chandler J Cochrane handbook for systematic reviews of interventions version 6.0 (updated July 2019). Cochrane 2019.

